# Lipocalin-7 Is a Matricellular Regulator of Angiogenesis

**DOI:** 10.1371/journal.pone.0013905

**Published:** 2010-11-09

**Authors:** Leslie J. Brown, Mariam Alawoki, Mary E. Crawford, Tiffany Reida, Allison Sears, Tory Torma, Allan R. Albig

**Affiliations:** Department of Biology, Indiana State University, Terre Haute, Indiana, United States of America; Institut Pasteur, France

## Abstract

**Background:**

Matricellular proteins are extracellular regulators of cellular adhesion, signaling and performing a variety of physiological behaviors such as proliferation, migration and differentiation. Within vascular microenvironments, matricellular proteins exert both positive and negative regulatory cues to vascular endothelium. The relative balance of these matricellular cues is believed to be critical for vascular homeostasis, angiogenesis activation or angiogenesis resolution. However, our knowledge of matricellular proteins within vascular microenvironments and the mechanisms by which these proteins impact vascular function remain largely undefined. The matricellular protein lipocalin-7 (LCN7) is found throughout vascular microenvironments, and circumstantial evidence suggests that LCN7 may be an important regulator of angiogenesis. Therefore, we hypothesized that LCN7 may be an important regulator of vascular function.

**Methodology and Principal Findings:**

To test this hypothesis, we examined the effect of LCN7 overexpression, recombinant protein and gene knockdown in a series of *in vitro* and *in vivo* models of angiogenesis. We found that overexpression of LCN7 in MB114 and SVEC murine endothelial cell lines or administration of highly purified recombinant LCN7 protein increased endothelial cell invasion. Similarly, LCN7 increased angiogenic sprouting from quiescent endothelial cell monolayers and *ex vivo* aortic rings. Moreover, LCN7 increased endothelial cell sensitivity to TGF-β but did not affect sensitivity to other pro-angiogenic growth factors including bFGF and VEGF. Finally, morpholino based knockdown of LCN7 in zebrafish embryos specifically inhibited angiogenic sprouting but did not affect vasculogenesis within injected embryos.

**Conclusions and Significance:**

No functional analysis has previously been performed to elucidate the function of LCN7 in vascular or other cellular processes. Collectively, our results show for the first time that LCN7 is an important pro-angiogenic matricellular protein of vascular microenvironments.

## Introduction

The control of angiogenesis is highly dependent on proper cues from the vascular microenvironment [1], [2]. Vascular microenvironments contain a host of factors including growth factors, cytokines and matricellular proteins which serve as critical extracellular integrators between structural and cellular activities [3]. By extension, perturbations of vascular microenvironments provoke major changes in vascular homeostasis including angiogenic activation and resolution [4]. In particular, tumor microenvironments are highly angiogenic and are characterized by altered expression of pro-angiogenic matricellular proteins. Understanding how the matricellular composition of tumor microenvironments is manipulated to enhance tumor angiogenesis is of central importance to understanding how to starve tumors of blood thus realizing the potential of anti-angiogenic approaches to cancer therapy.

Lipocalin-7 (LCN7, also Tinagl1-Tubulointerstitial nephritis antigen like 1, Tin-ag-RP-Tubulointerstitial nephritis antigen related protein, AZ-1-Adrenocortical zonation factor 1, and ARG1-Androgen regulated gene 1) is a matricellular protein that belongs to the diverse family of lipocalin proteins [5] and is highly conserved between species. LCN7 has been shown to be predominantly localized to vascular tissues in a variety of organ types. For example, LCN7 is abundant in vascular smooth muscle [6], lung capillary endothelium [7], uterine capillaries during the postimplantation period [8], vascular basement membranes of adrenocortical sinusoidal capillaries [9] and glomerular basement membranes [6]. In addition, LCN7 expression has been linked to a variety of physiological stimuli including testosterone signaling in the mouse prostate [10] and Hif1α activation in mouse brain [11]. Most recently it was also discovered that LCN7 is differentially regulated during angiogenesis [12]. Collectively, these findings demonstrate that LCN7 is found within vascular microenvironments and is differentially regulated during angiogenesis suggesting that LCN7 may be involved with regulation of vascular function. Despite this correlative evidence, there has been no direct attempt to assess the function of LCN7 in vascular function and angiogenesis.

Herein we show that LCN7 is a positive regulator of angiogenesis that increases endothelial cell invasion, angiogenic cell sprouting and sensitivity to TGF-β. Moreover, we demonstrate that LCN7 is important for early angiogenesis in zebrafish embryos. Collectively, these results indicate that LCN7 is a proangiogenic matricellular protein.

## Results

### Overexpression of LCN7 in endothelial cell lines

**Figure 1:**
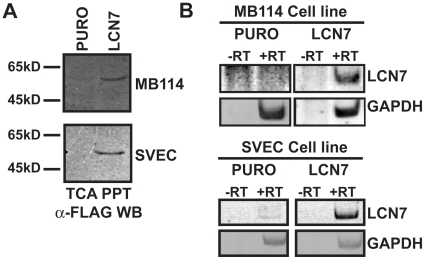
Overexpression of LCN7 in endothelial cells. Murine LCN7 cDNAs were C-terminally FLAG tagged and overexpressed from pMSCV retroviral vectors in MB114 and SVEC murine endothelial cell lines. **A**. Conditioned media from empty retroviral control cells (PURO) and LCN7-overexpressing cells was precipitated with TCA/DOC, fractionated through SDS-PAGE gels and western blotted with anti-FLAG monoclonal antibodies. Shown are representative images from a single experiment that was conducted twice with identical results. **B**. RT-PCR analysis was performed on either reverse transcribed (+RT) or non-reverse transcribed (-RT) RNA pools with oligonucleotides designed to amplify LCN7 (469 bp fragment) or GAPDH (237 bp fragment) as a loading control. Shown are representative images from a single experiment that was conducted three times with identical results.

LCN7 is consistently localized to vascular tissues [7], [8], [9], [13], has been linked to Hif1α activation in hypoxic brain damage [11] and was previously shown to be differentially expressed during endothelial cell angiogenesis on Matrigel matrices [12]. However, the functional role of LCN7 in vascular function and/or angiogenesis has not been addressed. Therefore, we set out to determine if LCN7 may be important for angiogenesis. To undertake this project, we first used a retroviral overexpresson strategy to drive LCN7 protein expression in mouse brain microcapillary endothelial (MB114) cells [14] and tumor derived SVEC4-10 (SVEC) endothelial cells [15]. The murine LCN7 cDNA was PCR amplified with oligonucleotides engineered to append a C-terminal FLAG epitope and subsequently cloned into a retroviral vector (pMSCV). MB114 and SVEC cell lines were transduced with LCN7 encoding pMSCV viral particles or empty, control viral particles. Polyclonal populations of cells harboring integrated sequences were selected with puromycin and tested for overexpression of FLAG-tagged LCN7. Conditioned media from the resulting overexpressing and control cell lines was collected, precipitated with TCA/DOC and analyzed by western blot with anti-FLAG M2 monoclonal antibodies. As shown in Figure 1A, both MB114 and SVEC cells expressed and secreted FLAG-tagged LCN7 protein. Overexpressed LCN7 migrated as a single 50 kD band suggesting that the overexpressed protein was not glycosylated as previously observed [16]. We attempted to compare the degree of LCN7 protein overexpression to basal LCN7 expression using commercially available LCN7 antibodies, however these antibody preparations demonstrated significant non-specific binding and failed to detect overexpressed protein much less endogenous LCN7 (data not shown). Instead, we used RT-PCR analysis to compare relative levels of LCN7 mRNA in control and overexpressing cells. We found that SVEC cells but not MB114 cells express low levels of endogenous LCN7 mRNA and that overexpression significantly increased LCN7 mRNA over basal levels in both cell lines (Fig. 1B). Collectively these results demonstrated that we successfully overexpressed LCN7 and allowed us to next examine the effect of LCN7 on several *in vitro* models of angiogenesis.

### Overexpression of LCN7 promotes pro-angiogenic endothelial cell behaviors

**Figure 2:**
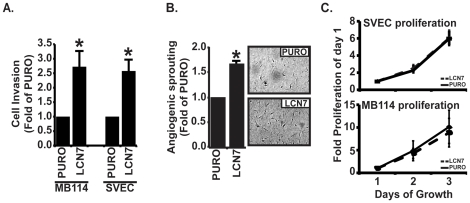
LCN7 increases endothelial cell angiogenic activities. **A**. Control (PURO) and LCN7-overexpressing endothelial cells cultured onto Matrigel coated trans-wells were induced to invade towards a 2% FBS gradient for 48 hours. Invading cells were stained with crystal violet and quantified by densitometry. Data is reported as average fold increase +/− SE of four independent experiments. **B**. Quiescent monolayers of control and LCN7-overexpressing MB114 endothelial cells were overlayed with a mixture of rat tail collagen and EGM2 media to induce angiogenic cell sprouting. Data was collected by manually counting the number of sprouting cells in 10 high power fields and is presented as the average fold increase +/− SE of three independent experiments. **C**. Control and LCN7-overexpressing cells seeded into tissue culture plates and cell proliferation was monitored daily for three sequential days with WST1. Data is represented as the fold increase +/− SE compared to each cell lines first measurement. For all panels, * indicates p<.05, Students t-test.

Angiogenesis is characterized by several distinct endothelial cell activities including endothelial cell proliferation, invasion through vascular basement membranes and sprouting from quiescent monolayers [17]. To measure the effect of LCN7 overexpression on these processes, we used a series of *in vitro* models that recapitulate these angiogenic activities. Transwell assays were first used to monitor the effect of LCN7 on endothelial cell invasion through artificial basement membranes (*i.e.* matrigel). As shown in Figure 2A, overexpression of LCN7 significantly increased both MB114 and SVEC endothelial cell invasion through Matrigel coated Boyden chambers as compared to respective control counterpart cells. We next examined the effect of LCN7 on anigogenic sprouting from quiescent MB114 endothelial monolayers that had been overlayed with rat tail collagen. This assay is designed to recapitulate the events that occur when quiescent vasculature encounters collagen I outside the vascular basement membrane during a wound healing process. As shown in Figure 2B, LCN7 overexpression significantly increased MB114 endothelial cell sprouting from quiescent monolayers into the collagen gel as compared to control cells. Finally, we used WST1 to measure the proliferation rates of control or LCN7-overexpressing MB114 and SVEC cells over a timecourse of three days in complete growth media. As shown in Figure 2C, overexpression of LCN7 did not have a significant impact on either MB114 or SVEC endothelial cell proliferation. Collectively, these results demonstrated for the first time that LCN7 promotes endothelial cell invasion and sprouting and suggest that LCN7 is a pro-angiogenic protein of the extracellular matrix (ECM).

### LCN7 promotes TGF-β signaling

**Figure 3:**
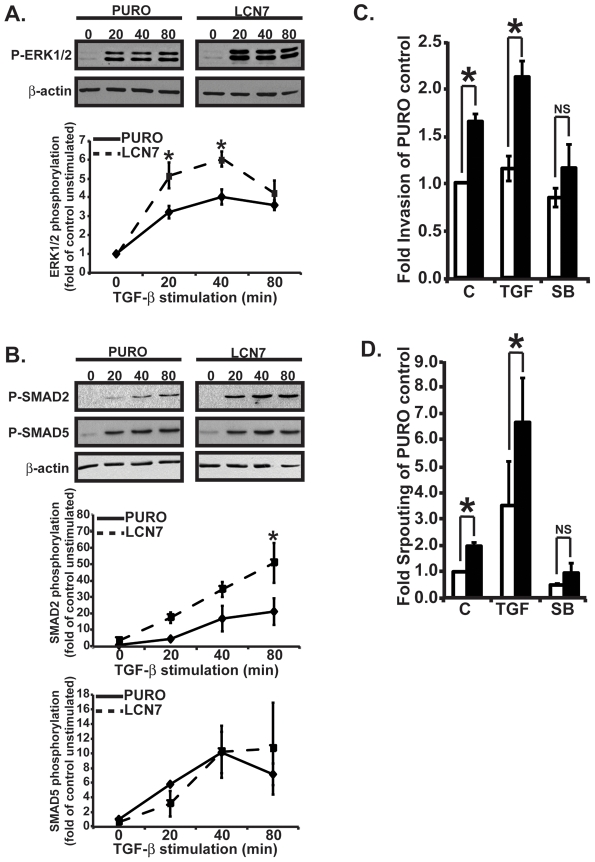
LCN7 sensitizes endothelial cells to TGF-β. **A**. Control and LCN7-overexpressing MB114 endothelial cells were serum starved then stimulated with 5 ng/ml of TGF-β1 for 0, 20, 40, or 80 minutes. Whole cell lysates were fractionated in SDS-PAGE and western blotted with antibodies to detect phosphorylated ERK1/2 MAPK (P-ERK1/2) or β-actin as a loading control. Western blot results from three independent experiments were quantified by densitometry, normalized to β-actin to control for loading differences, and are presented as the fold increase +/− SE of unstimulated control cells. **B**. Control and LCN7-overexpressing MB114 endothelial cells were stimulated as in A. Whole cell lysates were fractionated and western blotted with antibodies against phosphorylated SMAD-2 (P-SMAD2), phosphorylated SMAD5 (P-SMAD5), or β-actin. Western blot results from three independent experiments were quantified by densitometry, normalized to β-actin to control for loading differences, and are presented as the fold increase +/− SE of unstimulated control cells. **C**. Control or LCN7 overexpressing MB114 endothelial cells were induced to invade through matrigel coated transwells in the presence of 5 ng/ml TGF-β (TGF) or 10 µM TGF-β antagonist SB431542 (SB) and compared to unstimulated control (C) cells. Invading cells were stained with crystal violet and quantified by densitometry of stained invasion membranes. The experiment was performed three times and the resulting data is presented as the fold invasion of control cells +/− SE. **D**. Quiescent cultures of control or LCN7 overexpressing MB114 endothelial cells were overlayed with rat-tail collagen containing 5 ng/ml TGF-β (TGF) or 10 uM TGF-β antagonist SB431542 (SB) and compared to unstimulated control (C) cells. Data was collected by manually counting the number of sprouting cells in 10 high power fields and is presented as the average fold increase +/− SE of three independent experiments. * indicates p<.05, student's t-test.

Endothelial cell activation by growth factor stimulation is an early and critical aspect of angiogenesis activation. To begin dissecting the molecular mechanism by which LCN7 might promote angiogenesis, we first examined the activation ERK1/2 MAPK in LCN7-overexpressing cells and control cells after stimulation with various pro-angiogenic growth factors including VEGF, bFGF, and TGF-β. As shown in Figure 3, LCN7 overexpression significantly increased TGF-β induced phosphorylation of ERK1/2 MAPK. However, LCN7 did not have a significant effect on VEGF or bFGF induced ERK1/2 phosphorylation (data not shown). The precise mechanism by which TGF-β regulates angiogenesis remains the subject of much debate, however, it is generally accepted that TGF-β can have both pro- and anti-angiogenic activities and that these activities are mediated by differential activation of the ALK5-SMAD2/3 and ALK1-SMAD1/5/8 signal transduction pathways [18]. Therefore, we monitored TGF-β mediated phosphorylation of SMAD2 and SMAD5 in the presence or absence of LCN7 to further investigate the coupling of LCN7 and TGF-β in endothelial cells. As shown in Figure 3, LCN7 overexpression coupled TGF-β to the phosphorylation of SMAD2 but not to the phosphorylation of SMAD5. These results suggested that LCN7 may promote angiogenesis by enhancing endothelial cell sensitivity to TGF-β mediated ERK1/2 and SMAD2/3 signaling pathways.

To determine if enhanced TGF-β sensitivity in LCN7 overexpressing cells was functionally related to the pro-angiogenic activity of LCN7, we performed invasion and angiogenic cell sprouting assays in the presence of TGF-β or the TGF-β inhibitor SB431542. As shown in Figure 3C and 3D, the addition of TGF-β (5 ng/ml) to invading or sprouting cultures of control or LCN7 overexpressing MB114 cells provoked an increase in cellular invasion and sprouting. Consistent with our previous results, LCN7 overexpressing cells were more sensitive than control cells to the pro-invasive and pro-sprouting impact of TGF-β. Importantly, blockade of TGF-β signaling by 10 µM SB431542 reduced the enhanced invasion and sprouting activity of LCN7 overexpressing cells down to control levels. These results provided strong evidence that LCN7 promotes angiogenesis at least in part by sensitizing cells to the pro-angiogenic activity of TGF-β.

### Recombinant LCN7 mediates endothelial cell adhesion, invasion, and sprouting

**Figure 4:**
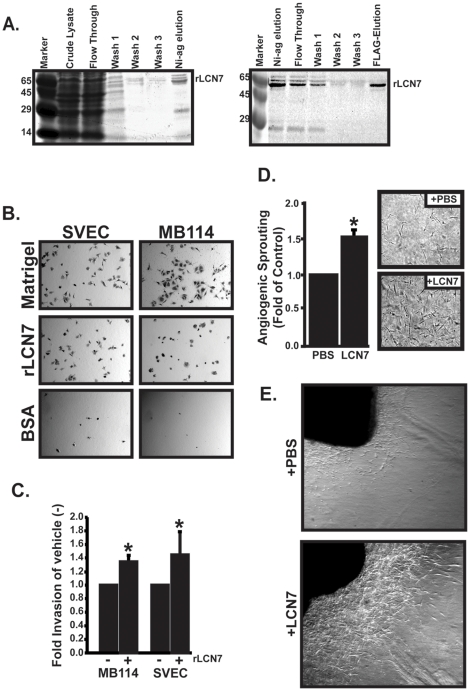
Recombinant LCN7 increases pro-angiogenic activities. **A**. Murine His-6 and FLAG-tagged LCN7 cDNA was expressed in *E. coli* and purified by sequential Ni^+^-agarose (left panel) and anti-FLAG M2 (right panel) affinity columns. Representative coomassie stained gels of the purification process are shown. LCN7 was purified to apparent homogeneity. **B**. Tissue culture wells were coated overnight with dilute Matrigel, 1 µg/ml of recombinant LCN7 (rLCN7), or 10 µg/ml of BSA, then all wells were blocked with 10 µg/ml BSA. Adherent SVEC and MB114 endothelial cells were stained with crystal violet. Shown are representative images of an experiment that was performed three times. **C**. MB114 and SVEC cells were cultured on Matrigel coated trans-wells with 1 µg/ml of rLCN7 or an equivalent volume of 1XPBS. Invading cells were stained with crystal violet and quantified by densitometry of stained invasion membranes. Results are depicted as the fold increase +/− SE compared to 1XPBS treated cells from a total of four independent experiments. **D**. Quiescent MB114 endothelial cells were overlayed with rat tail collagen containing 1 µg/ml of rLCN7 or an equivalent volume of PBS. After 48 hours, endothelial cell sprouting was quantified by manually counting the number of sprouting cells present in 10 high power microscopic fields. Results are depicted as the fold increase +/− SE compared to PBS treated cells from a total of three independent experiments. **E**. Sections of aorta from a single mouse were embedded into rat tail collagen containing either 1 µg/ml of rLCN7 or an equivalent volume of 1XPBS. Shown are representative images of sprouting cells after 4 days of incubation from a single experiment that was performed three times.

It was important to confirm that the pro-angiogenic effects of LCN7 were not dependent on retroviral overexpression and to confirm that LCN7 exhibits pro-angiogenic activity in other models of angiogenesis. To accomplish this, we used a combination of Ni+ agarose and anti-FLAG M2 affinity chromatography to purify murine recombinant 6-His and FLAG tagged LCN7 from E. coli cell lysates. As shown in Figure 4A, recombinant LCN7 preparations were free of contaminating proteins, had a molecular mass of approximately 50 kD, and were reactive with anti-FLAG antibodies. Having purified LCN7 protein, we set out to confirm the pro-angiogenic activity of LCN7 on endothelial cell adhesion, invasion and angiogenic sprouting.

Integrins serve as critical cell surface proteins that transmit signals from the extracellular matrix to the cell. Ligation of angiogenic integrins is an important aspect by which many matricellular proteins support endothelial cell adhesion and angiogenesis. LCN7 was previously shown to mediate adrenocortical, vascular smooth muscle and 293T cell adhesion [9], but endothelial cell adhesion to LCN7 has not previously been demonstrated. Therefore, we compared endothelial cell adhesion to either diluted matrigel as a positive control, BSA as a negative control, or purified LCN7 protein. Both MB114 and SVEC endothelial cells successfully adhered and spread out when cultured on both matrigel and recombinant LCN7 but failed to adhere to BSA (Fig. 4B).

Our previous results demonstrated that overexpression of LCN7 increased endothelial cell invasion and sprouting. Therefore, we also tested our recombinant preparations for similar pro-angiogenic activities. As shown in Figure 4C, addition of 1 µg/ml of purified LCN7 increased MB114 and SVEC invasion through matrigel coated boyden chambers. Moreover, inclusion of 1 µg/ml of recombinant LCN7 into collagen overlays also stimulated endothelial cell sprouting from quiescent MB114 monolayers. Finally, we tested the ability of recombinant LCN7 to induce angiogenic sprouting from *ex vivo* sections of mouse aortic rings. As shown in Figure 4E, collagen gels supplemented with 1 µg/ml of rLCN7 provoked more angiogenic sprouting from *ex vivo* aortic ring sections compared to aortic rings in PBS supplemented collagen. Collectively, these data confirmed that LCN7 promotes angiogenic cell behaviors in a variety of cell and culture systems. Based on these *in vitro* results, we set out to determine if LCN7 performs similar pro-angiogenic functions *in vivo*.

### Knockdown of LCN7 in zebrafish causes vascular defects

Zebrafish models of angiogenesis have become important tools to examine the function of angiogenic genes in *in vivo* settings. In particular, zebrafish strains expressing GFP from the endothelial specific *Fli1* promoter facilitate microangiogram analysis of even the earliest vascular beds in zebrafish embryos [19], [20]. Moreover, the ability to use anti-sense morpholinos (MO) to temporarily knockdown gene expression allows for the rapid analysis of gene function in the absence of targeted genes [21].

**Figure 5:**
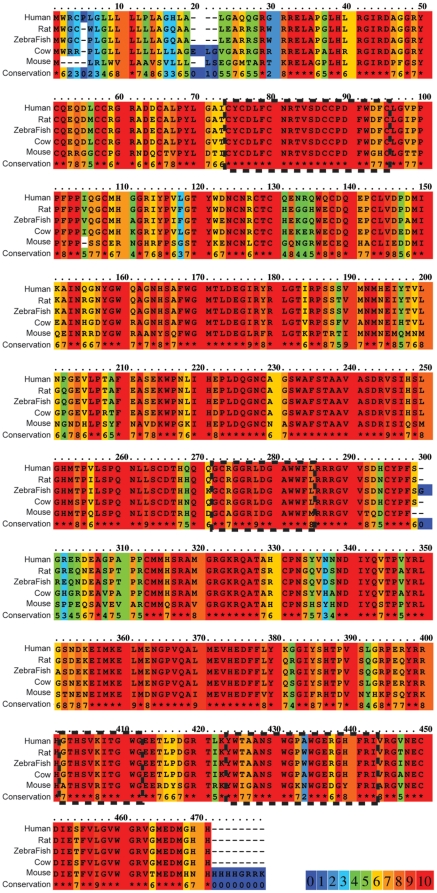
LCN7 is conserved between species. LCN7 protein sequences from human, rat, zebrafish, cow, and mouse were compared using PRALINE sequence alignment tool. The degree of conservation for each amino acid is indicated by color according to the key and in the “conservation” line of the alignment where * indicates 100% identity. Prosite analysis predicted the location of somatoamedin B (AA 74 to 95), lipocalin (AA 272 to 285), and cathepsin B (AA 402 to 452) and (AA 424 to 443) domains that are outlined with dashed lines. Overall, the alignment showed 79% sequence identity over the entire length of the alignment.

In order to determine if LCN7 is important for angiogenesis in developing zebrafish, we first performed a blast search of the full length murine LCN7 protein sequence against the zebrafish genomic database to identify the zebrafish LCN7 homolog. The zebrafish LCN7 gene encodes a 471 aa protein with predicted molecular weight of 53.9 kD. We used the PRALINE alignment tool (http://www.ibi.vu.nl/programs/pralinewww/) to construct a multiple sequence alignment of LCN7 proteins from human, rat, mouse, cow and fish (*D. rerio*). Multiple sequence comparison revealed that LCN7 was highly conserved between species over the entire length of the protein (Fig. 5). Features of the conserved alignment included N-terminal Somatomedin-B domain(s), inactive pro-cathepsin B-like domains and the signature lipocalin domain. Overall, the multiple sequence alignment revealed 79% sequence identity between all species over the length of the entire alignment.

**Figure 6:**
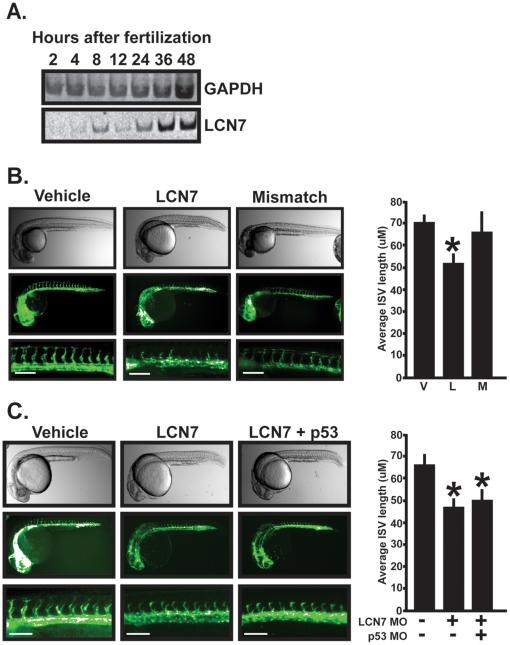
Morpholino knockdown in zebrafish embryos. **A**. LCN7 expression was detected in developing zebrafish embryos by RT-PCR analysis. cDNAs pools were generated from embryos ranging from 2 to 48 hours and subjected to RT-PCR analysis with either LCN7 or GAPDH specific oligonucleotides. **B**. Freshly fertilized zebrafish embryos between 1 to 8 cells were injected with morpholino vehicle (water +.1% phenol red) or equal volumes containing either 1 ng of LCN7 morpholino, or 1 ng of mismatched LCN7 control morpholino. Microangiogram analysis was performed approximately 28 hours later by detecting endothelial-specific expression of GFP. The accompanying graph depicts average ISV pixel length (converted to µM) as measured along the entire dorsal surface in each of five embryos in each experiment. Results shown are the average +/− SE of five independent experiments. Bar equals 100 µM. **C**. Freshly fertilized zebrafish embryos were injected with morpholino vehicle (water +.1% phenol red) or equal volumes containing 1 ng of LCN7 morpholinos or 1 ng of LCN7+1.5 ng of p53 morpholinos. ISV vessel length was measured as described above and results are presented as the average +/− SE of five independent experiments consisting of at least five individual fish per injection. For all panels, * indicates p<.05, students t-test. Bar equals 100 µM.

Before performing anti-sense morpholino knockdown of LCN7, it was first important to determine if LCN7 mRNA is present during early zebrafish development. To accomplish this, we used RT-PCR to monitor LCN7 mRNA in cDNA pools generated from 2 to 48 hour old embryos. As shown in Figure 6A, LCN7 mRNA was detectable within 8 hours after embryo fertilization and continued to be present over the remainder of the time course. Sequencing of the DNA fragments confirmed amplification of LCN7 cDNA (data not shown).

We designed a splice blocking anti-sense morpholino to transiently knockdown LCN7 expression in developing Fli1-GFP transgenic zebrafish embryos. Control LCN7 morpholinos were also designed with five mismatched bases and used as to control for possible non-specific affects caused by LCN7 morpholino injections. Fli1-GFP embryos (1 to 8 cells) were injected with 1 to 8 ng of either LCN7 antisense morpholino, LCN7 mismatched morpholino, or with equivalent volumes of morpholino vehicle alone (water +0.1% phenol red). As shown in Figure 6B, injection of 1 ng of LCN7 morpholino significantly delayed the development of intersegmental vessels (ISV) compared to embryos injected with either mismatched morpholinos or morpholino vehicle alone. Overall, approximately 24 hours after injection, ISV length in vehicle injected embryos was 70+/−3.7 µm. Injection of 1 ng of LCN7 MO reduced average ISV length to 51+/−4.8 µm. Injection of 1 ng of mismatched MO did not reduce ISV development compared to vehicle injected embryos. Moreover, and most likely as a consequence of delayed ISV development, dorsal longitudinal anastomatic vessel (DLAV) development was also delayed in LCN7 injected embryos. However, development of the aorta and the cardinal vein was unaffected indicating that angiogenesis, not vasculogenesis, was implicated with LCN7 deletion. Importantly, no defects in body patterning were obvious in embryos injected with up to 2 ng of morpholino solutions. Collectively, these results suggested that delayed ISV development was directly linked to LCN7 knockdown rather than to off-target effects of LCN7 morpholinos.

Although LCN7 knockdown appeared to produce specific effects on ISV development, injection of 4 ng or more of either LCN7 morpholinos or LCN7 mismatched control morpholinos also caused defects in body patterning and development (data not shown). Off-target morpholino effects are p53 dependent and simultaneous knockdown of p53 reverses these non-specific effects on zebrafish development [22]. Therefore, as an additional control for possible non-specific effects of LCN7 morpholino injections, we examined ISV development in LCN7 + p53 double knockdown embryos. To accomplish this, we injected 1 ng LCN7 morpholino together with 1.5 fold excess p53 specific morpholino as previously described [22]. As shown in Figure 6C, p53 co-knockdown failed to rescue the delay in ISV development produced by LCN7 knockdown. This result confirms that vascular defects induced by LCN7 knockdown are vascular-specific and not related to off target effects of LCN7 morpholinos. Collectively, these results indicate that LCN7 is essential for proper vascular function during angiogenesis *in vivo*.

## Discussion

Growth factors such as VEGF, bFGF, and TGF-β are some of the most potent regulators of angiogenesis. However, growth factor stimulation of endothelial cells alone is not sufficient to produce blood vessels with the physiological properties required for proper capillary function. This is illustrated by the leaky blood vessels observed in tumors that are stimulated by abnormally high secretions of pro-angiogenic growth factors such as VEGF [23]. Development and maintenance of a normal vascular system also requires input from structural and matricellular proteins of the vascular microenvironment such as LCN7. In fact, remodeling of structural and matricellular proteins within vascular microenvironments during angiogenesis or angiostasis serve a major role in controlling vascular function [2]. Because these proteins are so vital for proper vascular development, function and maintenance, their identification and characterization are necessary for pro- and anti-angiogenic therapies to achieve the great potential predicted by Judah Folkman and others [17], [24].

LCN7 has been shown to be expressed within vascular smooth muscle [6] and the endothelial cell lining of blood vessels [7], [9]. Furthermore, LCN7 was previously linked to Hif1α activation [11] and is differentially expressed during angiogenesis *in vitro* [12]. Such circumstantial observations spatially and temporally position LCN7 to be an important regulator of angiogenesis. Despite these observations, no functional analysis of LCN7 in angiogenesis or any other cellular process has previously been performed. In this study, we have used overexpression, recombinant protein and knockdown approaches combined with both *in vitro* and *in vivo* angiogenesis models to investigate the role of LCN7 in angiogenesis. Our results indicate that LCN7 mediates endothelial cell adhesion and is essential to angiogenesis in developing zebrafish embryos. Mechanistically, we found that LCN7 promotes angiogenesis by increasing endothelial cell invasion, sprouting and sensitivity to TGF-β.

Angiogenesis consists of a series of endothelial cell activities including response to pro-angiogenic growth factor signals, stimulation of cell proliferation, degradation of the vascular basement membrane, endothelial cell sprouting, migration and invasion of activated cells, formation of lumenized endothelial stacks and eventually, the development of anastomoses and capillary networks [25]. In this manuscript, we analyzed the function of LCN7 in several of these angiogenic stages. Our data demonstrates that both LCN7 overexpression and administration of highly purified recombinant LCN7 proteins increases invasion of two distinct endothelial cell lines. Moreover, we have also shown that overexpression of LCN7 and recombinant LCN7 promote endothelial cell sprouting from quiescent endothelial cell monolayers and from dissected *ex vivo* aortic rings.

The precise mechanism by which LCN7 promotes angiogenic activities remains largely unknown. However, our results provide the first evidence that LCN7 promotes angiogenesis by increasing endothelial cell sensitivity to TGF-β, a major determinant of pro-angiogenic signaling [18]. In support of this, we have shown that LCN7 increases ERK1/2 phosphorylation in TGF-β treated cells but not in cells treated with other angiogenic growth factors (VEGF or bFGF). We also demonstrated that the pro-angiogenic activity of TGF-β is enhanced in LCN7 overexpressing cell lines and that blockade of TGF-β signaling eliminates the increased invasion and angiogenic sprouting observed in LCN7 overexpressing MB114 endothelial cells.

Interestingly, both pro- and anti-angiogenic activities have been attributed to TGF-β. However, the mechanistic rationale for this dichotomy are the subject of ongoing debate. One perspective on this debate is that TGF-β activation of SMAD2/3 via the ALK5 receptor is coupled to angiogenesis activation while TGF-β activation of SMAD1/5/8 via the ALK1 receptor mediates angiogenesis resolution [26], [27], [28], [29]. Consistent with this model, we have found that LCN7 increases coupling of TGF-β to SMAD2 but not SMAD5. However, another perspective on this debate claims that it is activation of ALK1 and SMAD1/5/8 by TGF-β that mediates angiogenic activation and moreover that SMAD1/5/8 activation suppresses the angiostatic effects of SMAD2/3 activation [30], [31], [32]. Although it is beyond the scope of this report, it will be critical in future research to more closely examine the link between LCN7, TGF-β and angiogenesis to gauge the importance of TGF-β signaling to LCN7's pro-angiogenic activities and to provide insight into the controversy regarding the role of TGF-β in angiogenesis.

Our data also determined that LCN7 mediates endothelial cell adhesion. Consistent with this finding, Li et al [9] previously demonstrated that LCN7 promotes vascular smooth muscle cell adhesion by binding to α1, α2, α5 and β1 integrin receptors. Although the identity of integrin receptors involved in endothelial-LCN7 adhesion remain unknown, all of the afore mentioned integrin subunits are expressed in endothelial cells and each of the possible combinations (*i.e.* α1β1, α2β1, and α5β1) are known regulators of angiogenesis [33]. Therefore it is likely that in addition to sensitizing endothelial cells to TGF-β, LCN7 may also promote angiogenesis by mediating ligation of pro-angiogenic integrins. Future studies will focus on identifying LCN7 binding integrins in endothelial cells and determining the importance of these interactions to LCN7's pro-angiogenic activities.

Finally, LCN7 contains two protein domains that may provide additional hints at the protein's pro-angiogenic activity. First, LCN7 is a member of the lipocalin family of extracellular matrix proteins which is very diverse and serves a wide range of functions [5]. Of the lipocalins, lipocalin-2 (LCN2, also neutrophil-gelatinase associated lipocalin, NGAL) is most relevant to this study since LCN2 suppresses angiogenesis in pancreatic cancer [34]. Given the small size of the lipocalin domain and the divergence of function between LCN2 and LCN7, it seems unlikely that any general conserved angiogenic function can be assigned to lipocalin proteins. LCN7 also contains cathepsin B-like domains. Cathepsin B is a secreted protease of the cathepsin family which is important for proteolytic processing of extracellular matricies, cellular invasion, migration and are linked to various human pathologies of the cardiovascular and other systems [35]. However, the cathepsin domains of LCN7 are not active [6], [10]. This observation has led to the hypothesis that LCN7 may serve as an inhibitor of cathepsin activity and block cell invasion and migration. Our results do not support this hypothesis since we find that LCN7 increases endothelial cell invasion and sprouting.

In summary, we have identified and characterized LCN7 as a novel matricellular regulator of angiogenesis. Although we determined that LCN7 promotes endothelial cell invasion, sprouting and angiogenesis *in vivo*, there remain a significant number of questions that remain to be addressed regarding the biological function of LCN7. For instance, future studies need to be performed to 1.) more precisely define the mechanism by which LCN7 promotes angiogenesis, 2.) describe the impact of LCN7 on additional cell types present in vascular microenvironments, and 3.) determine if LCN7 is an important regulator of angiogenesis in various human diseases characterized by insufficient or excessive angiogenesis.

## Materials and Methods

### Ethics Statement

Animal studies were performed in accordance with the animal protocol procedures approved by the Institutional Animal Care and Use Committee of Indiana State University (protocol #1-19-2008:AA and 11-08-2007:AA).

### Cell biological assays (proliferation, invasion, sprouting assays)

Angiogenic sprouting of MB114 cells in collagen matrices was performed as described previously [36]. Briefly, control- or LCN7-overexpressing cells (400,000 cells/well) were seeded onto 6-well plates and allowed to grow to confluence. Confluent monolayers were overlayed with 2 ml of solidified rat tail collagen containing 10%FBS and 1X MB114 cell media. Sprouting was allowed to proceed for 5 days, whereupon sprouting cells were visualized by carefully focusing a 20X objective just above the plane of quiescent endothelium to identify cells that sprouted from the monolayer and entered the collagen matrix. The number of invading angiogenic sprouts was quantified under a light microscope by determining the average number of sprouts present in 10 independent fields/well.

Proliferation assays were performed by seeding 1000 control or LCN7-overexpressing MB114 or SVEC endothelial cells into 96-well plates in 100 µl of complete growth media. Cell proliferation was monitored daily for 3 days by adding 10 µl of WST1 for 2 hours after which absorbance at OD450 in the conditioned media was measured.

Invasion assays were performed using a modified Boyden-chamber assay as described previously [36], [37]. Briefly, upper chambers (8 µm pore, 24-well format; BD Biosciences) were coated with 100 µl of diluted Matrigel [1∶50 in serum-free DMEM (SFM)], which was evaporated overnight at room temperature. Subsequently, control or LCN7-overexpressing MB114 or SVEC cells were washed twice in SFM containing 0.1% BSA (SFM/0.1% BSA) and seeded in upper chambers at a density of 100,000 cells/well. Endothelial cell invasion was stimulated by addition of 2% serum to lower chambers, and was allowed to proceed for 48 h at 37°C. Invading endothelial cells were washed, fixed in 95% ethanol, and stained with crystal violet. The number of invading endothelial cells was determined by densitometric analysis of scanned invasion membranes.

Aortic ring assays were performed by first dissecting the aorta from freshly sacrificed C57BL/6 mice and slicing the aorta into approximately 1 mm thick rings. Up to 10 aortic rings isolated from each animal were equally divided and half of each group was imbedded into collagen gels containing 1 µg/ml of recombinant LCN7 while the other half of each group was imbedded into collagen gels containing an equal volume of PBS. Collagen gels were formed by isolating rat tail collagen as previously described [38], mixing purified collagen 1∶1 with EGM2 media (clonetics) and overlaying the solidified collagen gels with EGM2 media.

### Recombinant LCN7 production and purification

Murine LCN7 was amplified and prepared for gateway cloning into pDEST42 by a two-stage PCR reaction that was accomplish using oligo sets that were designed to delete the first 18 amino acids and add an ATTB1 site to the 5′ end while the FLAG epitope tag and ATTB2 sequences were appended to the 3′ end of the protein. First 5′ oligo: AAAGCAGGCTTCACCATGGAGGCCCGGCGGAGTCGT. Second 5′ oligo: GGGGACAAGTTTGTACAAAAAAGCAGGCTTCACCATG. First 3′ oligo: CTTATCGTCGTCATCCTTGTAATCGTGGTGCCCCATGTCCTC. Second 3′ oligo: GGGGACCACTTTGTACAAGAAAGCTGGGTCCTTATCGTCGTCATC. The complete PCR product was cloned into an entry vector with BP clonase (Invitrogen), subsequently cloned into pDEST42 with LR clonase (Invitrogen) and eventually sequenced in its entirety. To produce recombinant LCN7 in BL21-DE3 cells, LCN7 expression was induced with IPTG and the resulting protein purified from bacterial cell lysates first on a Ni-agarose affinity column and second on an anti-FLAG M2 affinity column using standard procedures. Protein purity was monitored by coomassie staining of SDS-PAGE gels.

### Morpholinos and morpholino injections

The LCN7 specific anti-sense morpholino (Genetools) (5′TTAAACTCACTGGGAGGGTAGGGAG) was designed to interfere with splicing at the LCN7 exon1 – intron1 junction. The mismatched control LCN7 morpholino (5′GTAAACGCACTGCGAGGGTTGGGAA ) was identical to the specific LCN7 morpholino except for the presence of 5 mismatched bases (underlined) that disrupt efficient binding to the LCN7 exon1 – intron1 splice junction. Morpholinos were dissolved in water and diluted 1∶1 into 0.1% phenol red/water injecting solution. Injections into 1 to 8 cell *Fli1-*GFP embryos ranged from 1 to 8 ng of LCN7 morpholino in a total volume of 0.3 to 0.6 nl per embryo. Approximately 28 hours after fertilization, embryos were sedated in tricaine and monitored for vascular phenotypes on a Nikon SMZ-1500 fluorescent dissecting microscope. Morpholinos directed against p53 were synthesized according to previously published work [22].

### Plasmids and Retroviral Infections

A retroviral LCN7 vector was constructed by PCR amplifying the full-length murine LCN7 cDNA from clone ID 3596560 (open biosystems). To accomplish this, oligos were designed such that an ATTB1 site was appended to the 5′ end and the FLAG epitope tag and ATTB2 sequences were appended to the 3′ end through two rounds of sequential PCR with the following oligos. First forward LCN7 oligo: AAAGCAGGCTTCACCATGTGGGGATGTTGGCTG. Second forward LCN7 oligo: GGGGACAAGTTTGTACAAAAAAGCAGGCTTCACCATG. First reverse LCN7 oligo: CTACTTATCGTCGTCATCCTTGTAATCGTGGTGCCCCATGTCCTC. Second reverse LCN7 oligo: GGGGACCACTTTGTACAAGAAAGCTGGGTCCTACTTATCGTCGTCATC. The PCR product was cloned by BP clonase into an entry vector and subsequently cloned with LR clonase into a custom made pMSCV retroviral vector featuring the ATTR1-CM^r^-ccdB-ATTR2 cassette blunt cloned into the HPA1 sites of pMSCV-PURO. Clones were selected by ampicillin resistance, tested for inserts by restriction digest with EcoR1 and Xho1, and sequenced (Functional Genomics).

Control (i.e., pMSCV-IRES-GFP) and LCN7 retroviral supernatants were produced and infected into murine brain microvascular MB114 endothelial cells and SVEC tumor derived endothelial cells as described previously [36], [37], [38], [39]. Infected cells were isolated by puromycin selections (4 µg/ml) to yield stable polyclonal populations of control or LCN7-overexpressing cells. LCN7 expression was confirmed by TCA/DOC precipitation of conditioned media with anti-FLAG M2 monoclonal antibodies.

### Reverse-Transcription PCR

Total RNA was collected from either zebrafish embryos or from mammalian endothelial cells using Tri Reagent (Sigma) according to the manufacturer's recommendations. cDNA was generated from 1 µg of total RNA that was reverse transcribed in 10 µl reactions according to the manufacturer's recommendations (iScript, Biorad), diluted to 100 µl, and used for PCR (2.5 µl cDNA/25 µl reaction). Oligonucleotides used to amplify zebrafish LCN7 were as follows: Forward: 5′ TGCCTTACCTGGACACCATCTGTTA, Reverse: 5′ ACCGGTGGCCATTTCTTTCACAAG. Oligonucleotides used to amplify zebrafish GAPDH were as follows. 5′ AGGCTTCTCACAAACGAGGACACA, Reverse 5′ ATCAATGACCAGTTTGCCGCCTTC. Oligonucleotides used to amplify mouse LCN7 were as follows. 5′ TGCCTTACCTGGACACCATCTGTTA, reverse 5′ ACCGGTGGCCATTTCTTTCACAAG. Oligonucleotides used to amplify mouse GAPDH were as follows. 5′ GACAATGAATACGGCTACAGCAAC, Reverse 5′ GTGCAGCGAACTTTATTGATGGTA. All RT-PCR products were sequenced at least once to confirm amplification of correct target sequences.
